# Components of Metabolic Syndrome as Risk Factors for Hearing Threshold Shifts

**DOI:** 10.1371/journal.pone.0134388

**Published:** 2015-08-06

**Authors:** Yu-Shan Sun, Wen-Hui Fang, Tung-Wei Kao, Hui-Fang Yang, Tao-Chun Peng, Li-Wei Wu, Yaw-Wen Chang, Chang-Yi Chou, Wei-Liang Chen

**Affiliations:** 1 Department of Family and Community Medicine, Division of Family Medicine, Tri-Service General Hospital; and School of Medicine, National Defense Medical Center, Taipei, Taiwan, Republic of China; 2 Department of Family and Community Medicine, Division of Geriatric Medicine, Tri-Service General Hospital; and School of Medicine, National Defense Medical Center, Taipei, Taiwan, Republic of China; 3 Graduate Institute of Clinical Medical, College of Medicine, National Taiwan University, Taipei, Taiwan, Republic of China; 4 Department of surgery, Division of plastic surgery, Tri-Service General Hospital; and School of Medicine, National Defense Medical Center, Taipei, Taiwan, Republic of China; 5 Graduate Institute of Medical Sciences, National Defense Medical Center, Taipei, Taiwan, Republic of China; Sun Yat-sen University, CHINA

## Abstract

**Background:**

Hearing loss was a common, chronically disabling condition in the general population and had been associated with several inflammatory diseases. Metabolic syndrome, which was associated with insulin resistance and visceral obesity, was considered a chronic inflammatory disease. To date, few attempts had been made to establish a direct relationship between hearing loss and metabolic syndrome. The aim of the present study was to investigate the relationship between metabolic syndrome and hearing loss by analyzing the data in the reports of the National Health and Nutrition Examination Survey 1999–2004.

**Methods:**

This study included 2100 participants aged ≤ 65 years who enrolled in the National Health and Nutrition Examination Survey (1999–2004). We examined the relationship between the presence of different features of metabolic syndrome in the participants and their pure-tone air-conduction hearing thresholds, including low-frequency and high-frequency thresholds.

**Results:**

After adjusting for potential confounders, such as age, medical conditions, and smoking status, the participants with more components of metabolic syndrome were found to have higher hearing thresholds than those with fewer components of metabolic syndrome (p < 0.05 for a trend). The low-frequency hearing threshold was associated with individual components of metabolic syndrome, such as abdominal obesity, high blood pressure, elevated triglycerides, and a low level of high-density lipoprotein cholesterol (HDL-C) (p < 0.05 for all parameters).

**Conclusions:**

The results indicated that the presence of a greater number of components of metabolic syndrome was significantly associated with the hearing threshold in the US adult population. Among the components of metabolic syndrome, the most apparent association was observed between low HDL and hearing loss.

## Introduction

Hearing loss is a common, chronically disabling condition in the general population [1], and its prevalence is increasing due to exposure to noisy environments, the use of listening devices and the growth of the elderly population [2]. Hearing loss affects communication and quality of life [3], both of which have been significantly associated with the cognitive impairment and independence of elderly people [4, 5]. However, the pathophysiological mechanisms underlying hearing loss are complicated. Multiple risk factors contribute to hearing loss, including inflammatory processes, systemic diseases, genetic susceptibility, and oxidative stress [6, 7]. A relationship between hearing loss and inflammatory diseases, such as diabetes [8] and cardiovascular disease[9], has been demonstrated. Notably, several components of metabolic syndrome, such as elevated blood pressure and dyslipidemia, have been correlated with the risk factors for hearing loss [10, 11]. The results of a prospective longitudinal registry study of the birth weight of 245,000 Swedish conscripts support the theory that sensorineural hearing loss might be a new clinical feature of metabolic syndrome [12].

Metabolic syndrome, which consists of the cluster of elevated blood pressure, atherogenic dyslipidemia, glucose intolerance and central obesity [13], has been strongly associated with an increased risk of cardiovascular disease, coronary heart disease, diabetes and all causes of mortality [14, 15]. Among the five components of metabolic syndrome, the prevalence of insulin resistant and abdominal obesity has increased during the last decade [16]. Chronic inflammation, which is associated with insulin resistance and visceral obesity, is found to be an important factor in the pathophysiology of metabolic syndrome[17, 18]. To date, few attempts have been made to establish a direct relationship between hearing loss and metabolic syndrome. A retrospective study of 181 participants who suffer from a sudden onset of hearing loss shows that metabolic syndrome is an independent risk factor [19]. Therefore, we hypothesize that the presence of a greater number of features of metabolic syndrome would be associated with an impaired hearing threshold. The aim of the present study is to investigate the relationship between metabolic syndrome and hearing loss by analyzing the data in the reports of the National Health and Nutrition Examination Survey 1999–2004.

## Methods

### Ethics statement

The NHANES study protocol was approved by the National Center for Health Statistics (NCHS) Institutional Review Board (IRB). Because our analysis exclusively used de-identified data, it was exempt from IRB review.

### Study populations

NHANES was a multi-stage cross-sectional survey that was designed to assess the health and nutritional status of the civilian noninstitutionalized United States (U.S.) population. The survey, which was conducted by the Centers for Disease Control and Prevention (CDC) and the National Center for Health Statistics (NCHS), included an initial extensive household interview and a subsequent physical examination at a specially equipped Mobile Examination Center (MEC). Trained examiners collect pertinent information during the home interview, including age, gender, race, results of physical examinations, and medical history. Since 1999, NHANES had been a continuous annual survey rather than a periodic survey, and the survey data had been released every 2 years. Detailed survey operations manuals, consent documents, and brochures for NHANES 1999–2004 were available on the NHANES website [20–22].

Pertinent information, including demographic data, the results of the examination, laboratory results, questionnaire contents, and audiometric measurements, were collected from 3 NHANES datasets (1999–2000, 2001–2002, and 2003–2004). The population examined in the present study consisted of adults aged 20–65 years. We excluded participants for whom data were lacking regarding the components of metabolic syndrome, the household interview, the results of laboratory and clinical examinations, and the audiometric measurements, and those with current noise exposure.

### Audiometric measurements

Among the participants chosen using the 1999–2004 NHANES datasets, half of the participants aged 20–69 years were randomly assigned to the Audiometry Examination Component of the study. Among these populations, eligible individuals who were unable to remove their hearing aids or who could not tolerate the testing headphones were excluded. The audiometric examinations were conducted in a sound-isolated room by health technicians trained by an audiologist who was certified by the National Institute for Occupational Safety and Health. An audiometer (model AD226; Interacoustic, Assens, Denmark) equipped with standard TDH-39P headphones (Telephonics Corp, Farmingdale, NY, USA) and insertional earphones (model EARTone 3A; Etymotic Research, Elk Grove Village, IL, USA) was used for the audiometric examinations [23]. The pure-tone air-conduction hearing thresholds for each ear were measured at 0.5, 1, 2, 3, 4, 6, and 8 kHz over an intensity range of –10 to 120 dB HL. The pure-tone average (PTA) at 0.5, 1, and 2 kHz was defined as the low-frequency PTA (low-PTA), whereas the PTA at 3, 4, 6, and 8 kHz was defined as the high-frequency PTA (high-PTA). The hearing thresholds for the ear with worse perception were chosen for the regression analysis.

### Definition of metabolic syndrome

Metabolic syndrome was defined by the revised National Cholesterol Education Program’s Adult Treatment Panel III (NCEP: ATP III) as the presence of three or more of the following characteristics: (1) abdominal obesity: waist circumference of ≥102 cm in men and ≥88 cm in women; (2) hypertriglyceridemia: ≥150 mg/dL (≥1.69 mmol/L);(3) a low level of high-density lipoprotein cholesterol (HDL-C): <40 mg/dL (<1.03 mmol/L) in men and <50 mg/dL (<1.29 mmol/L) in women; (4) elevated blood pressure: systolic blood pressure (SBP) ≥130 mm Hg or diastolic blood pressure (DBP) ≥85 mmHg; and (5) elevated fasting glucose: ≥100 mg/dL (≥5.6 mmol/L)[18].

### Covariates

Demographic information was collected, including age, sex, race, smoking status, and medical history. A computer-assisted personal interviewing (CAPI) method was used. Race/ethnicity was classified as non-Hispanic white, non-Hispanic black or others. Smoking status was classified based on the answer to the question “Do you now smoke cigarettes?”. Diabetes was defined according to a self-report of a physician's diagnosis, a fasting glucose level ≥ 126 mg/dl, a random glucose level ≥ 200 mg/dl, or the use of diabetic medications (including injected insulin or an oral hypoglycemic agent). Co-morbidities, including stroke, heart disease, and chronic kidney disease, were ascertained by self-reports. The presence of heart disease was defined according to whether the participant had experienced or had been told he had experienced a myocardial infarction, congestive heart failure or angina. The use of ototoxic medication was defined according to the self-reported use of loop diuretics, aminoglycoside, antineoplastic drugs or nonsteroidal anti-inflammatory drugs. Abnormal otoscopy was defined according to any abnormal result of a brief otoscopic screening exam of the ear canals and eardrums before audiometry, including abnormal excessive or impacted ear cerumen, physical abnormalities, or collapsing external ear canals. Tympanometry was performed using an acoustic impedance tympanometer (Earscan, Micro Audiometric, Murphy, NC). Abnormal tympanometry was defined according to middle ear peak pressure ≤ −150 daPa or compliance ≤ 0.3 ml. Waist circumference was measured at the high point of the iliac crest at minimal respiration to the nearest 0.1 cm. Biochemical analyses of total cholesterol, triglyceride and HDL-C (Hitachi 704 Analyzer) serum contents were performed in the Lipoprotein Analytical Laboratory of Johns Hopkins University, Baltimore, Maryland. Serum glucose was determined using an enzymatic assay (Cobas Mira assay). Three and sometimes four blood pressure measurements of all of the eligible individuals were performed in the MEC and during the home examinations using a mercury sphygmomanometer. The averages of all of the systolic and diastolic blood pressure values were used. Serum uric acid was measured using a Hitachi 737 automated multichannel chemistry analyzer (Boehringer Mannheim Diagnostics, Indianapolis, IN, USA). All of the protocols utilized standardized methods with documented accuracy according to the reference methods of the CDC.

### Statistical analyses

All of the statistical analyses were conducted using the SPSS (Version 18.0 for Windows, SPSS, Inc., Chicago, IL, USA) procedure Complex Samples to incorporate the sample weights and adjust for clusters and the strata of the complex sample design. Two-sided p values less than 0.05 were considered to indicate significant differences. The values for quantitative parameters are expressed as the mean value and standard deviation (SD), whereas qualitative data are presented as numbers and percentages. Log transformation was performed to normalize the distributions of the PTA hearing threshold values. The effect of all of the components of metabolic syndrome on the PTA hearing threshold for PTA was examined using a linear regression model.

An extended-model approach was used to adjust the covariates: Model 1 = age, gender, and race/ethnicity; Model 2 = Model 1+ uric acid level; Model 3 = Model 2+ smoking status, abnormal otoscopy, abnormal tympanometry, current use of ototoxic agent, history of diabetes mellitus, heart disease and stroke. The components of metabolic syndrome were treated as continuous variables ranging from 1 to 5 to allow the assessment of the P-values for the trends and therefore the associations across the existence of increasing numbers of metabolic syndrome components and PTA hearing thresholds.

## Results

### Characteristics of the study population

The study population consisted of 2026 participants, including 609 with metabolic syndrome and 1417 without metabolic syndrome. The clinical characteristics of the study population categorized according to metabolic syndrome are summarized in Table 1. The participants with metabolic syndrome were older, had a higher hearing threshold in both ears, higher uric acid levels, higher frequencies of diabetes, heart disease, stroke, current use of an ototoxic agent, abnormal tympanometry and fewer smokers than those without metabolic syndrome (Table 1).

**Table 1:** Characteristics of participants with or without metabolic syndrome.

Variables	Metabolic syndrome		
	Yes (n = 609)	No (n = 1417)	P value
**Continuous variables, mean ± SD**			
Age (year)	47.39±12.21	39.20±12.93	<0.001
Fasting glucose (mg/dl)	118.00±50.78	93.20±15.20	<0.001
Waist (cm)	106.01±13.90	91.67±13.09	<0.001
Triglyceride (mg/dl)	238.96±269.39	114.26±68.22	<0.001
HDL-C (mg/dl)	43.26±11.35	56.73±15.76	<0.001
Systolic blood pressure (mmHg)	129.34±18.13	116.38±14.98	<0.001
Diastolic blood pressure (mmHg)	75.88±12.52	70.60±11.09	<0.001
Uric acid (mg/dl)	5.73±1.44	5.05±1.36	<0.001
Right ear			
Low-PTA (dB)	14.33±11.06	10.67±9.22	<0.001
High-PTA (dB)	25.31±18.52	18.62±17.04	<0.001
Left ear			
Low-PTA (dB)	14.10±10.95	10.45±8.96	<0.001
High-PTA (dB)	25.68±18.75	19.45±17.42	<0.001
Worse ear			
Low-PTA (dB)	16.68±12.36	12.85±9.94	<0.001
High-PTA (dB)	29.11±20.01	22.18±18.40	<0.001
**Categorical variables (%)**			
Male	44.7	48.6	0.059
Race			
Non-Hispanic white	46.3	48.5	0.002
Non-Hispanic black	16.9	21.9	0.002
Other	36.8	29.6	0.002
Smoking	49.8	55.6	0.055
Ever had diagnosis			
Diabetes	15.7	2.1	<0.001
Heart disease	5.6	2.2	<0.001
Chronic kidney disease	2.0	1.2	0.130
Stroke	2.8	1.3	0.015
Ototoxic medication (current use)	13.0	8.3	0.001
Abnormal tympanometry	11.9	8.6	0.018
Abnormal otoscopy	20.7	19.2	0.237

Definition of abbreviations: HDL-C = high-density lipoprotien cholesterol

Low-PTA = Pure tone average at low frequencies

High-PTA = Pure tone average at high frequencies

### Association between the metabolic syndrome components and hearing thresholds

The results obtained from modeling the association between the presence of metabolic syndrome components and the hearing thresholds are presented in Table 2 and Table 3. As shown in these tables, there was a strong linear increase in the hearing threshold with the presence of metabolic syndrome components and an increasing number of components of metabolic syndrome. After adjusting for other covariates in Model 3, the β coefficients of the hearing thresholds of participants with ≤2, 3, and ≥4 features of metabolic syndrome were 0.048, 0.040, and 0.077 for the high-PTA values and 0.011, 0.044, and 0.084 for the low-PTA values (p value for the trends of 0.004 and 0.009, respectively). A high triglyceride level and low HDL-C level was significantly associated with both high-PTA and low-PTA hearing thresholds in the fully adjusted models (p<0.05), and abdominal obesity and high blood pressure were significantly associated with only an increased low-PTA hearing threshold in the fully adjusted low-PTA models (p<0.05). A high level of serum glucose was the only component of metabolic syndrome that was not significantly associated with an increased low-PTA hearing threshold in all of the low-PTA adjusted models.

**Table 2:** Regression coefficients of number of metabolic syndrome for hearing threshold.

Variables		High-PTA				Low-PTA			
		Unadjusted	Model 1	Model 2	Model 3	Unadjusted	Model 1	Model 2	Model 3
Presence of metabolic syndrome	β	0.144	0.049	0.058	0.052	0.122	0.080	0.087	0.075
	(95%CI)	(0.112,0.176)	(-0.002,0.075)	(0.003,0.083)	(-0.002,0.079)	(0.094,0.151)	(0.011,0.091)	(0.015,0.097)	(0.006,0.089)
	P value	<0.001	0.066	0.033	0.060	<0.001	0.012	0.008	0.024
Number of metabolic syndrome									
≦2	β	0.076	0.038	0.043	0.048	0.058	0.005	0.009	0.011
	(95%CI)	(0.039,0.113)	(-0.014,0.073)	(-0.009,0.078)	(-0.015,0.089)	(0.025,0.091)	(-0.042,0.049)	(-0.039,0.052)	(-0.037,0.053)
	P value	<0.001	0.178	0.124	0.087	0.001	0.880	0.793	0.738
3	β	0.147	0.033	0.043	0.040	0.122	0.043	0.050	0.044
	(95%CI)	(0.105,0.190)	(-0.021,0.081)	(-0.012,0.091)	(-0.015,0.089)	(0.085,0.160)	(-0.019,0.086)	(-0.014,0.093)	(-0.018,0.088)
	P value	<0.001	0.243	0.134	0.160	<0.001	0.207	0.151	0.198
≧4	β	0.199	0.070	0.080	0.077	0.167	0.089	0.096	0.084
	(95%CI)	(0.153,0.245)	(0.015,0.124)	(0.023,0.134)	(0.020,0.133)	(0.127,0.207)	(0.019–0.133)	(0.024,0.139)	(0.013,0.130)
	P value	<0.001	0.013	0.006	0.008	<0.001	0.008	0.006	0.016
P for trend		<0.001	0.007	0.002	0.004	<0.001	0.006	0.003	0.009

Model 1 adjust for age, gender, race

Model 2 adjust for age, gender, race, and uric acid

Model 3 adjusted for age, gender, race, and uric acid, smoking status, abnormal otoscopy, abnormal tympanometry, current used of ototoxic agents, Ever had diagnosis: diabetes, heart disease, stroke

Definition of abbreviations: Low-PTA = Pure tone average at low frequencies, High-PTA = Pure tone average at high frequencies

**Table 3:** Regression coefficients of component of metabolic syndrome for hearing threshold.

Component of metabolic syndrome		High-PTA				Low-PTA			
		Unadjusted	Model 1	Model 2	Model 3	Unadjusted	Model 1	Model 2	Model 3
Abdominal obesity	β	0.044	0.013	0.016	0.018	0.065	0.050	0.046	0.054
	(95%CI)	(0.024,0.064)	(-0.016,0.034)	(-0.015,0.036)	(-0.014,0.038)	(0.048,0.083)	(0.003,0.056)	(0.000,0.055)	(0.005,0.060)
	P value	<0.001	0.487	0.422	0.375	<0.001	0.029	0.051	0.022
Elevated BPBP	β	0.198	0.038	0.038	0.035	0.138	0.056	0.054	0.050
	(95%CI)	(0.176,0.219)	(0.000,0.055)	(0.000,0.056)	(-0.003,0.053)	(0.119,0.157)	(0.006,0.066)	(0.005,0.065)	(0.003,0.062)
	P value	<0.001	0.054	0.052	0.077	<0.001	0.017	0.023	0.032
Elevated triglyceridetriglyceride	β	0.073	0.069	0.076	0.072	0.066	0.086	0.089	0.079
	(95%CI)	(0.042,0.104)	(0.013,0.084)	(0.018,0.090)	(0.015,0.087)	(0.038,0.093)	(0.016,0.090)	(0.018,0.093)	(0.012,0.086)
	P value	<0.001	0.007	0.003	0.005	<0.001	0.005	0.004	0.010
Low level of HDL-C	β	0.041	0.059	0.042	0.052	0.046	0.076	0.074	0.056
	(95%CI)	(0.020,0.063)	(0.017,0.065)	(0.017,0.066)	(0.012,0.061)	(0.027,0.065)	(0.021–0.073)	(0.019,0.072)	(0.008,0.061)
	P value	<0.001	0.001	0.001	0.004	<0.001	<0.001	0.001	0.010
Elevated fasting glucose	β	0.179	0.003	0.006	0.004	0.107	0.002	0.002	-0.008
	(95%CI)	(0.149,0.210)	(-0.036,0.040)	(-0.034,0.043)	(-0.037,0.042)	(0.079,0.134)	(-0.039,0.041)	(-0.039,0.042)	(-0.046,0.036)
	P value	<0.001	0.919	0.819	0.893	<0.001	0.957	0.940	0.805

Model 1 adjust for age, gender, race

Model 2 adjust for age, gender, race, and uric acid

Model 3 adjusted for age, gender, race, and uric acid, smoking status, abnormal otoscopy, abnormal tympanometry, current used of ototoxic agents, Ever had diagnosis: diabetes, heart disease, stroke

Definition of abbreviations: BP = blood pressure, HDL-C = high-density lipoprotien cholesterol, Low-PTA = Pure tone average at low frequencies, High-PTA = Pure tone average at high frequencies

## Discussion

By analyzing the data for a representative sample of the US population, we found that the presence of metabolic syndrome, as defined by the revised ATP III criteria, was significantly associated with both high- and low-frequency hearing thresholds, as determined in audiometric examinations of the adult population. We observed a positive relationship between the hearing thresholds and an increased number of metabolic syndrome components. Notably, among all of the metabolic syndrome components, a low HDL level and a high triglyceride level demonstrated a stronger association with the increased hearing thresholds.

Whereas a link between dyslipidemia and auditory function was plausible, little empirical evidence supported a direct relationship. The first study of this topic supporting the possibility that dyslipidemia may be associated with reduced hearing was published in 1964[24]. More recently, studies had shown that an elevated serum triglyceride level was significantly associated with the level of auditory function[11, 25], which was consistent with the findings of our study. Notably, the results of our study indicated that among the five metabolic syndrome components, a reduced level of HDL-C had the strongest association with an increased hearing threshold, which was consistent with the findings of previous studies [9, 26]. In several experimental studies, the investigators attempted to determine the pathophysiologic mechanisms underlying the effect of dyslipidemia on hearing function. Electron microscopic examinations revealed vacuolar edema and degeneration of the stria vascularis and the capillary vessels surrounding the vascular stria in guinea pigs that were fed a lipid-rich diet [27, 28]. Moreover, nitric oxide (NO), which was produced in the cochlear blood vessels, contributed to the regulation of cochlear blood flow, and its level might be related to different forms of hearing disorders [29, 30]. Reactive oxygen species formation in the inner ear, which caused cellular death, vasoconstriction and reduced cochlear blood flow, also played a key role in hearing loss [7, 31]. HDL-C was reported to have an anti-apoptotic, anti-oxidant, anti-inflammatory, and NO-promoting effect [32, 33]. It was tempting to speculate that a reduced level of HDL was significantly associated with an increased hearing threshold through the mediation of multiple pathways that were involved in the pathogenesis of hearing loss.

Obesity was found to be associated with hearing loss in both humans and animals [34, 35]. Moreover, it was proposed that central obesity, as well as an increased waist circumference or content of visceral adipose tissue, was significantly associated with an increased hearing threshold after adjusting for body mass index [36, 37]. In the present study, we also observed that an increased waist circumference was significantly associated with an increase high-PTA threshold. In previous studies concerning the relationship between the components of metabolic syndrome and chronic kidney disease or lung functionality, waist circumference played a similarly important role among the five metabolic syndrome components [38, 39]. The level of adiponectin, an adipocytokine that was secreted by adipose tissue, was found to be inversely correlated with waist circumference, insulin resistance, and inflammatory status [40, 41]. A cross-sectional study conducted by Hwang et al (2011) indicated that the plasma adiponectin concentration correlated negatively with the hearing thresholds, particularly the high-frequency threshold [42]. Based on the above-mentioned rationale, the plasma adiponectin level would decrease in parallel with the increase in waist circumference, which played a critical role in hearing loss.

Our study had a few limitations. NHANES was designed as a cross-sectional study in which the hearing thresholds and presence of metabolic syndrome components were determined at one point rather than repeatedly over a long-term observational period. Thus, it may be not possible to determine the directionality of the established associations. Another limitation of our study was the absence of questions regarding congenital, genetic or childhood hearing in the NHANES dataset. Additionally, we cannot exclude the effect of recall bias on the medical history data.

## Conclusion

The results of our study indicated that the presence of a greater number of metabolic syndrome components was significantly associated with the hearing threshold of the US adult population. Among the metabolic syndrome components, the association between a low level of HDL-C and hearing loss was most apparent. Given the inevitable risk factors, our findings suggest that the resolution of metabolic syndrome and reduction of the severity of the components of metabolic syndrome might contribute to a reduced risk of hearing loss.
